# Aggressive Palliative Surgery in Metastatic Phyllodes Tumor: Impact on Quality of Life

**DOI:** 10.4103/0973-1075.68402

**Published:** 2010

**Authors:** A S Kapali, M Singh, SVS Deo, N K Shukla, Dillip K Muduly

**Affiliations:** Department of Surgical Oncology, BR Ambedkar Institute Rotary Cancer Hospital (BRAIRCH), All India Institute of Medical Sciences (AIIMS), New Delhi, India

**Keywords:** Hamilton depression rating scale, Palliative surgery, Phyllodes tumor, Quality of life

## Abstract

Metastatic phyllodes tumor has very few treatment options. Phyllodes tumor in metastatic setting has limited role of surgery, radiotherapy and chemotherapy or combined treatment. Most of the patients receive symptomatic management only. We present a case of metastatic phyllodes tumor managed with aggressive margin negative resection of primary tumor leading to palliation of almost all the symptoms, which eventually led to improved quality of life and probably to improved survival. The improved quality of life was objectively assessed with Hamilton depression rating scale. Surgery may be the only mode of palliation in selected patients that provides a better quality of life and directly or indirectly may lead to improved survival.

## BACKGROUND

The phyllodes tumor includes a group of lesions of varying malignant potential, ranging from completely benign tumors to fully malignant sarcomas. They are rare with an incidence of 0.3-1.0% of all breast neoplasms.[1] Phyllodes tumors are classified as benign, borderline, or malignant on histological criteria of which stromal overgrowth is most important.[2] Surgery with margin negative excision is the primary mode of management in benign and malignant tumors.[3,4] Radiotherapy is beneficial as adjuvant modality after surgery in high risk individuals in gaining local control of disease in borderline and malignant phyllodes tumors.[5,6] Systemic therapy does not appear to have much benefit in phyllodes tumor.[7,8] Malignant phyllodes can spread to distant sites, most commonly to lungs. In metastatic settings all the three modalities surgery, chemotherapy and radiotherapy have very limited role, so majority of patients just receive supportive treatment. We highlight a case of metastatic phyllodes with large fungating growth in which aggressive margin negative surgical resection of primary tumor was done leading to marked symptomatic improvement in patient quality of life. This improvement was analyzed with Hamilton depression rating scale (HDRS) an objective depression scale with decrease in score after surgery compared to the score before surgery.[7]

## CASE REPORT

**Figure 1:**
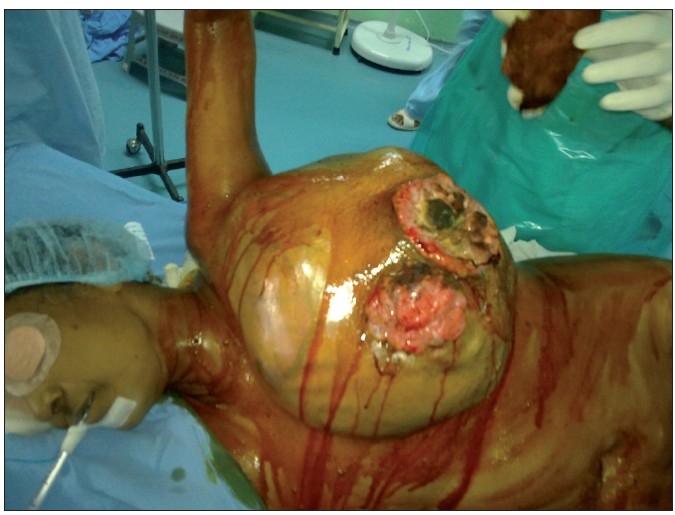
Preoperative figure showing huge tumor in the left breast

A 57-year-old postmenopausal woman presented with a 3 month history of recurrent lump (Past history of marginal excision outside twice, 12 months and 4 months before) in left breast of 30 × 25 × 15 cm in size with bossalated surface and profuse foul smelling discharge from necrotic surface ulcer and off and on fever with occasional chills [Figure 1]. Patient was a school teacher and had difficulty in performing her routine work due to the tumor weight, foul smell, discharge and ill health. She had no co morbidities or family history of any malignancy. Previous histopathology report documented it to be of malignant phyllodes tumor but no surgical details were available. Her HDRS score was calculated on admission. HDRS’s main purpose was to assess severity of, and change in, depressive symptoms. It is the most widely used clinician-administered depression assessment scale. It has 17 questions on mood and other symptoms which are scored. It consists of a set of 17 questions on depressed mood, feelings of guilt, suicide, insomnia in early and middle of night, insomnia early in the morning, work and activities, retardation, agitation, anxiety psychic, anxiety somatic, somatic symptoms of gastrointestinal, general somatic, genital symptoms, hypochondrias, loss of weight and insight which are given scores.[9] This provides with an objective score which is done before and after treatment to quantify the improvement in depressive symptoms. Patient’s score was HDRS 15 on admission to the hospital.

**Figure 2:**
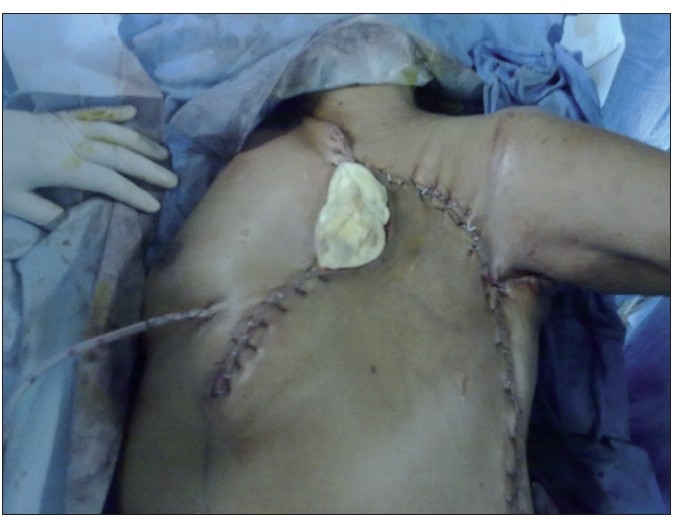
Postoperative image after radical resection and reconstruction

**Figure 3:**
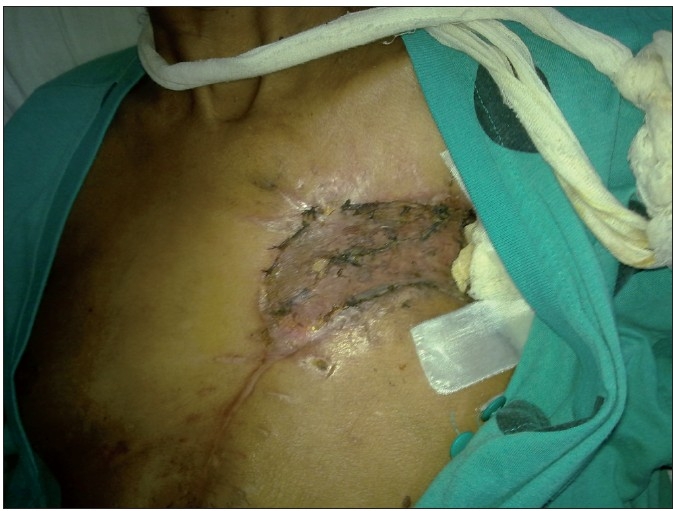
Follow-up image after one month of surgery

On examination, patient was anemic with average general condition. Her core biopsy was suggestive of malignant phyllodes tumor. CECT of thorax was suggestive of solitary pulmonary metastasis in lower zone of right lung. She was planned for a staged surgery (wide local excision and local flap cover followed by metastatectomy in second stage). As per planning, she underwent radical wide excision with 2 cm margin which included total mastectomy with excision of pectoralis major and lateral border of latissimus dorsi muscle. Medial based thoraco-abdominal rotation advancement flap used for local wound cover [Figure 2]. Post operative period was complicated by loss of 2 × 3 cm of the TE flap superomedialy which was managed with debridement and split thickness skin grafting [Figure 3]. Patient was re-evaluated after 3 weeks of radical wide excision for status of pulmonary metastasis which had increased in number and size suggestive of progressive disease. Despite having progressive disease the patient’s general condition had markedly improved, she had gained appetite and weight. Patient’s HDRS score was 3 at the time of discharge from surgical ward. Histology of the primary tumor showed a 28 × 22 × 12 cm solid necrotic spindle cell tumor with hemorrhage involving overlying skin and nipple areolar complex with 30 mitoses per 10 high power field with infiltrating margin, consistent with malignant phyllodes tumor. All margins including deep resected margin were negative. She was started with ifosfamide and cisplatin as a palliative regime for adjuvant chemotherapy. There is no local recurrence till date (6 months).

## DISCUSSION

The present patient is a case of metastatic phyllodes with all the features of poor prognosis. Hawkins *et al*. showed that four features (high mitotic count, stromal overgrowth, severe nuclear pleomorphism, and infiltrating margins) were predictors for the development of metastases. Of these, stromal overgrowth is the most reliable predictor. Tumor size was also an important prognostic variable in determining the overall survival. Fou *et al*. suggested a 75% mortality in metastatic phyllodes with mitotic index >10 per hpf.[10] This patient had a large tumor (30 cm) with >30 mitosis per hpf and infiltrating margins indicating a highly aggressive disease. Suzuki *et al*. from Japan have shown just 11% 2 year survival in their review of 15 patients of metastatic phyllodes tumor, and in their case report, the patient survived just 5 months.[8] Asogolu *et al*. in 2006 reported that, no clinical trials were available to assess the best treatment regimen for metastatic phyllodes, experience with systemic therapy for metastatic phyllodes tumor is anecdotal.[11] Chemotherapy may occasionally afford some palliation, and its combination with doxorubicin has been shown by Hawkins *et al*. to even cause complete remission but there are a few case report.[11,12] Ifosfamide is thought to be the most active agent.[11] There is no demonstrated role for hormonal therapy. Surgery has been shown to optimize local control in metastatic breast cancer and is hypothesized to improve survival,[13] but its role in metastatic phyllodes is not known. Radiotherapy according to Barth RJ *et al*. was effective in improving local control after surgery in malignant phyllodes, but its role in metastatic setting has not been described.[5]

Metastatic phyllodes tumor with such dismal prognosis, the tendency is to offer only symptomatic and supportive care with no significant role of surgery in such setting. But in the present case surgical resection relieved the patient of pain (i.e. Total pain which means psychosocial, physical and spiritual distress combine to affect the patient). Surgical resection has lead to -

- Improvement in psychological state of patient as shown by HDRS score which decreased to 3 from 15, also there was a generalized mood upliftment.
- Patient was relieved of her physical pain and her chronic inflammatory state due to the presence of tumor, which was necrotic with infection and discharging foul smelling purulent sanguinous fluid. She became afebrile with increased appetite and improved her nutritional status and gained weight.
- Improvement also occurred in social and spiritual aspects. Patient on her follow up visit described the joy of being back to her social life and she felt herself being freed of a long standing curse.

Surgery till date is considered to be futile in metastatic malignancies, but the impact of local control achieved by surgery in metastatic phyllodes tumor has lead to a better quality of life and is hypothesized to improve survival. The present case also highlights the fact that surgery should aggressive, the patient had underwent surgery twice before she presented to us which seemed to be marginal excision with futile results, it was only after radical surgery that adequate local control was achieved and quality of life improved. Steyaert *et al*. have improved survival with radical resection in metastatic breast cancer,[14] these facts suggest that surgery whenever done in breast malignancies should be aggressive and margin negative whether it is curative or palliative.

Hypothesis: Aggressive surgical resection in the patient discussed may lead to improved survival by improvement in physical and mental well being leading to improved nutritional and immunological status to be eligible for chemotherapy. The role of chemotherapy in improving survival in metastatic phyllodes is still to be defined but drugs such as ifosfamide and doxorubicin may have a role.

- With the advent of cancer stem cells and their role in metastasis,[14] the removal of primary tumor which may be the source of these stem cells is suggested to improve survival.

## CONCLUSION

Surgical resection may not have a proven role in metastatic phyllodes tumor treatment. But in some situations, it may be the only modality for palliation and provide a better quality life to the patient and directly or indirectly may lead to improved survival.

"Everyone has a right to good quality of life, however short it may be."
